# Causal relationships between gut microbiota, immune cell, and Non-small cell lung cancer: a two-step, two-sample Mendelian randomization study

**DOI:** 10.7150/jca.92699

**Published:** 2024-02-04

**Authors:** Jiabin Chen, Xuzhou Yu, XiaoYu Wu, Kequn Chai, Sheng Wang

**Affiliations:** 1 The Second Clinical Medical College, Zhejiang Chinese Medicine University, Hangzhou, Zhejiang 310012, China.; 2 Respiratory Department, Zhejiang Jinhua Guangfu Cancer Hospital, Jinhua Zhejiang 310053, China.; 3 Department of Oncology, Tongde Hospital of Zhejiang, Hangzhou, Zhejiang 310012, China.

**Keywords:** Gut microbiota, Immune cell, Non-small cell lung cancer, Mendelian randomization

## Abstract

**Background:** Regulating the immune system is a crucial measure of gut microbiota (GM) that influences the development of diseases. The causal role of GM on Non-small cell lung cancer (NSCLC) and whether it can be mediated by immune cells is still unknown.

**Methods:** We performed a two-step, two-sample Mendelian randomization study with an Inverse variance weighted (IVW) approach to investigate the causal role of GM on NSCLC and the mediation effect of immune cells between the association of GM and NSCLC.

**Results:** MR analyses determined the protective effects of 6 genera on NSCLC (Bacteroides, Roseburia, Alistipes, Methanobrevibacter, Ruminococcus gauvreauii group, and Peptococcus). In addition, 38 immune cell traits were suggestively associated with NSCLC. Of note, the mediation MR illustrated the causal role of Genus-Peptococcus on NSCLC (Total effect IVW: OR = 0.790, 95% CI [0.657, 0.950], P = 0.012) was to a large proportion mediated by CD45 on HLA DR^+^ CD4^+^ in TBNK panel (-034 (95% CI [-0.070, -0.005]; P = 0.037), accounting for 14.4% of Total effect).

**Conclusion:** The study suggested a causal relationship between GM and NSCLC, which may be mediated by immune cells.

## Introduction

In most countries, lung cancer (LC) remains the deadliest type of malignant tumors and one of the most commonly diagnosed cancers 1. Non-small cell lung cancer (NSCLC), the primary histological subtype of LC, accounts for more than 80% of all LC cases 1,2. Although improvements in therapeutic strategies of LC, such as targeted therapy and immunotherapy, the disease still has a dismal overall prognosis— the 5-year survival rate is only 25% 3,4. Even worse, over 70% of NSCLC patients already have locally advanced or distant metastasis at the initial diagnosis, with a lower 5-year overall survival rate (<10%) 5. Therefore, understanding the mechanism of occurrence and development of NSCLC and developing novel, safe, and efficient treatment methods for NSCLC are urgently needed.

In recent years, gut microbiota (GM) has attracted considerable attention from researchers as the second genome in human beings and performs a vital role in human health 6,7. Emerging evidence has shown that the differential composition and distribution of GM in various diseases and GM dysregulation may contribute to the development of diseases, including NSCLC 8,9. In addition, a cohort study revealed that the use of antibiotics would increase the risk of NSCLC and affect the prognosis, due to the imbalance of intestinal homeostasis 10-12.

Regulating metabolism, endocrine, inflammation, and immune system are the main measures of GM to influence the development of diseases 13,14. GM could enhance the immune function of the system and increase the efficacy of immunotherapy on gastrointestinal cancers by altering the local environment of the intestinal mucosa and intestinal-associated lymphoid tissue 15. The immune system is the crucial factor in the development, infiltration, and metastasis of NSCLC. Immune cells recognize and eliminate malignant cells to function immune surveillance 16. However, malignant cells can evade immune surveillance by various mechanisms in certain situations 16.

In epidemiology, Mendelian randomization (MR) is an analytical tool to explore etiological inferences and deduce relationships between risk factors (exposure) and outcomes 17. Previously, much evidences have illustrated the role of GM in NSCLC and the effect of GM on the immune system. Nevertheless, whether GM could affect the progression of NSCLC by regulating the immune system is still unknown. The study aimed to investigate the causal role of GM on NSCLC and whether it can be mediated by immune cells. Firstly, we collected single nucleotide polymorphism (SNP) data as instrumental variables (IVs) of exposure. Then, a comprehensive two-sample MR analysis was performed to estimate the causal role of GM and immune cells signatures on NSCLC. Finally, we explored the effects of GM on immune cell signatures and calculated the proportion of GM's effect on NSCLC mediated by immune cell signatures to assess whether GM could affect the progression of NSCLC by regulating the immune system.

## Materials and methods

### Study design

**Figure 1:**
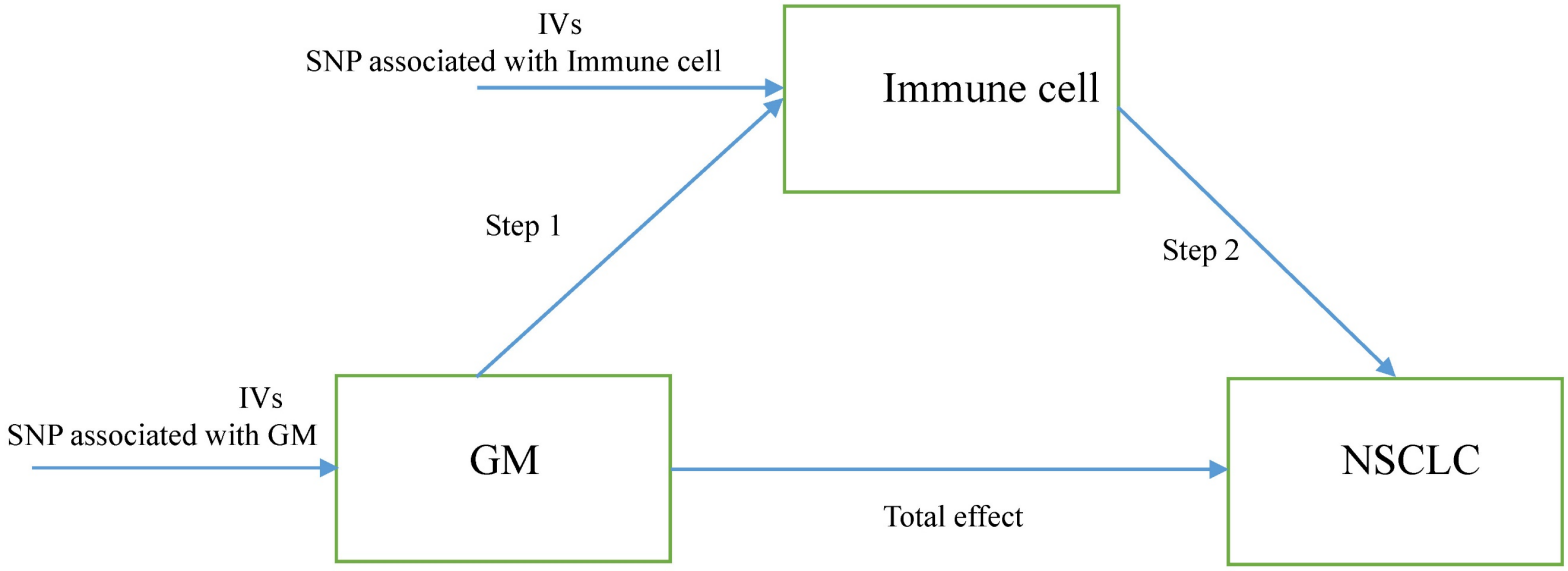
The study design. A two-step Mendelian randomization study of GM on NSCLC mediated by immune cell. GM: Gut microbiota; NSCLC: Non-small cell lung cancer; IVs: Instrumental variables.

Herein, we performed a two-step MR to determine the relation of GM to the genetically predicted risk of NSCLC and whether immune cell signatures could mediate this association. The first step is evaluating the causal effect of GM and immune cell signatures on NSCLC with a two-sample MR and screening out GM and immune cell signatures highly related to the risk of NSCLC. The second step is evaluating the causal effect of the filtered GM on the filtered immune cells' signatures and calculating the proportion of mediation of each mediator for GM's effect on NSCLC. In addition, the research objects should not overlap, meaning SNP represented exposure and outcome should be from different research sources. The study design was presented in Figure 1.

### Data sources

Summary statistic data for GM at the genus level were collected from MiBioGen (https://mibiogen.gcc.rug.nl/), which included 119 genera (Ebi-a-GCST90016908 to Ebi-a-GCST90017118). Data for NSCLC was derived from Finn-b-C3_LUNG_NONSMALL, including 1627 NSCLC cases and 217165 control cases 18. Data for 731 immune cell traits (Ebi-a-GCST0001391 to Ebi-a-GCST0002121) were from the GWAS Catalog (Genome-wide association studies, https://gwas.mrcieu.ac.uk/) 19. 731 immune cell traits could be divided into six panels: B cells, CDCs, mature stages of T cells, monocytes, myeloid cells, TBNK (B cells, natural killer cells, T cells), and Treg panels. In addition, 731 immune cell traits included absolute cell (AC) counts (n=118), relative cell (RC) counts (n=192), median fluorescence intensities (MFI) reflecting surface antigen levels (n=389), and morphological parameters (MP) (n=32). All participants in the study were from Europe.

### Genetic instrumental variables (IVs) selection

The filter condition of SNPs as IVs for GM and immune cell traits was set to p < 1e×10^-5^ in accordance with previous researches 20. SNPs as IVs of NSCLC were determined with a stricter value (p <5e×10^-8^). We clumped all those genetic variants with the threshold: R^2^ < 0.001 within 10000 kb clumping distance. The F-statistics was applied to screen for SNPs in the end. The calculation method of the F-statistics was β divided by the square of the standard error, and the cut-off value was 10. We searched the obtained SNPs to find potential confounders and bypassing (e.g. Age, sex, race, other disease) with PhenoScanner V2 (http://www.phenoscanner.medschl.cam.ac.uk/) 21.

### Statistical analysis

All statistical analysis were implemented with R 4.3.1 (https://www.r-project.org). The packages "TwoSampleMR," package "VariantAnnotation" package, and "ieugwasr" were used to conduct two-sample MR analysis. Among five methods ("MR Egger" 22, "Weighted median" 23 "Inverse variance weighted (IVW)" 24, "Simple mode" 25 and "Weighted mode" 23) in MR analysis, IVW was the primary method for causal estimation due to it was the most precise and robust way. P < 0.05 was considered to have a significant association between exposure and outcome. The statistical power was analyzed using an online web tool (http://cnsgenomics.com/ shiny/mRnd/) 26. Cochran's Q statistic based on IVW and MR Egger methods was utilized to assess the degree of heterogeneity. We took advantage of the MR-Egger intercept test and MR pleiotropy residual sum and outlier method (MR-PRESSO) to detect the pleiotropy with the package: "MR-PRESSO." Furthermore, MR-PRESSO was also applied to correct horizontal pleiotropy via outlier removal and assess significant differences before and after outlier correction. Leave-one-out analysis was used to explore the influence of possible outlying genetic variants. The indirect effect of GM on NSCLC risk via potential mediator was evaluated with the "product of coefficients" method. Standard errors for the indirect effects were determined with the delta method.

## Results

### Total effect of GM on NSCLC

**Figure 2:**
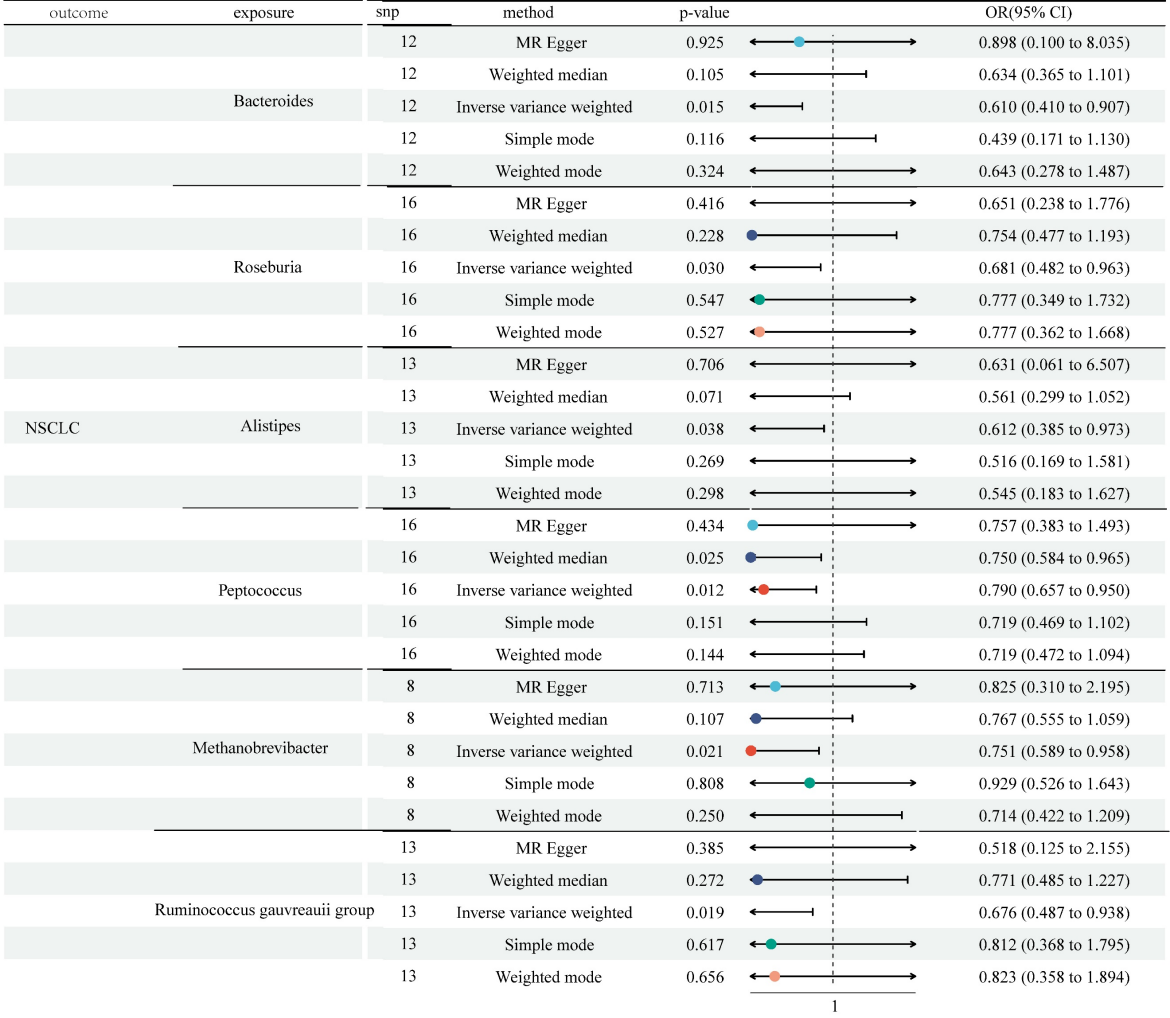
MR analysis showed the causality of 6 genera on NSCLC were significant (Bacteroides, Roseburia, Alistipes, Methanobrevibacter, Ruminococcus gauvreauii group, and Peptococcus). CI: Confidence Interval; GM: Gut microbiota; MR: Mendelian randomization; OR: odds ratio; SNP: Single nucleotide polymorphism.

The study selected 1531, 13318, and 9 SNPs as IVs for 119 genera, 731 immune cell traits, and NSCLC (Table S1). The two-sample MR analysis demonstrated the causality of 6 genera on NSCLC (Figure 2 and Table S2). Although other methods did not reflect statistical significance, the IVW approach revealed increased Bacteroides were associated with a lower risk of NSCLC (odds ratio (OR) = 0.610, 95% Confidence Interval (CI) [0.410, 0.907], P = 0.015). Similar results were observed in Roseburia (OR = 0.681, 95% CI [0.482, 0.963], P = 0.030), Alistipes (OR = 0.612, 95% CI [0.385, 0.973], P = 0.038), Methanobrevibacter (OR = 0.751, 95% CI [0.589, 0.958], P = 0.021), and Ruminococcus gauvreauii group (OR = 0.676, 95% CI [0.487, 0.938], P = 0.019). Both the IVW method and Weighted median method illustrated the protective effects of Genus-Peptococcus on NSCLC (IVW: OR = 0.790, 95% CI [0.657, 0.950], P = 0.012; Weighted median: OR = 0.750, 95% CI [0.584, 0.965], P = 0.025). Cochran's Q statistic, MR-Egger intercept test, and MR-PRESSO indicated no heterogeneity or horizontal pleiotropy in this MR analysis (Table S6 and Table S7). Further, no single SNP seriously violated GM's overall impact on NSCLC in the leave-one-out sensitivity analysis (Figure S1).

### Effect of immune cell traits on NSCLC

**Figure 3:**
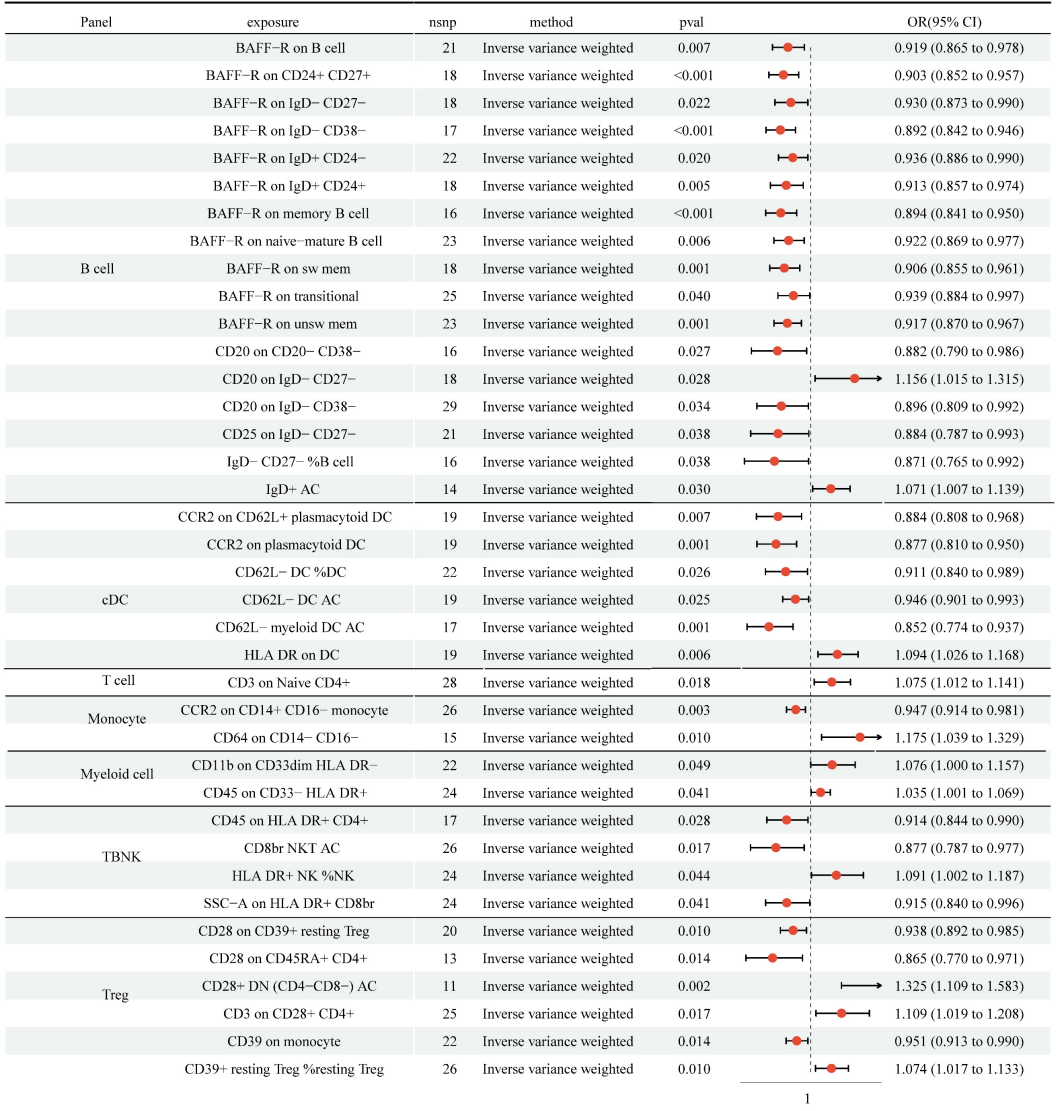
MR analysis showed 27 immune cell traits had protective effects on NSCLC and 11 immune cell traits had disadvantageous effects on NSCLC. CI: Confidence Interval; GM: Gut microbiota; MR: Mendelian randomization; OR: odds ratio; SNP: Single nucleotide polymorphism.

We detected protective effects of 27 immune cell traits on NSCLC with the IVW approach (Figure 3, Table S3). In addition, genetically predicted 11 immune cell traits would increase the risk of NSCLC. Cochran's Q statistic-derived p values based on IVW and MR Egger methods were more significant than 0.05, hinting no apparent heterogeneity was found (Table S6). The MR-Egger intercepts test and MR-PRESSO were not statistically significant, indicating no horizontal pleiotropy (Table S7). The leave-one-out analysis showed that removing a particular SNP would not change the causal estimates (Figure S2).

### Effect of GM on immune cell traits

**Figure 4:**
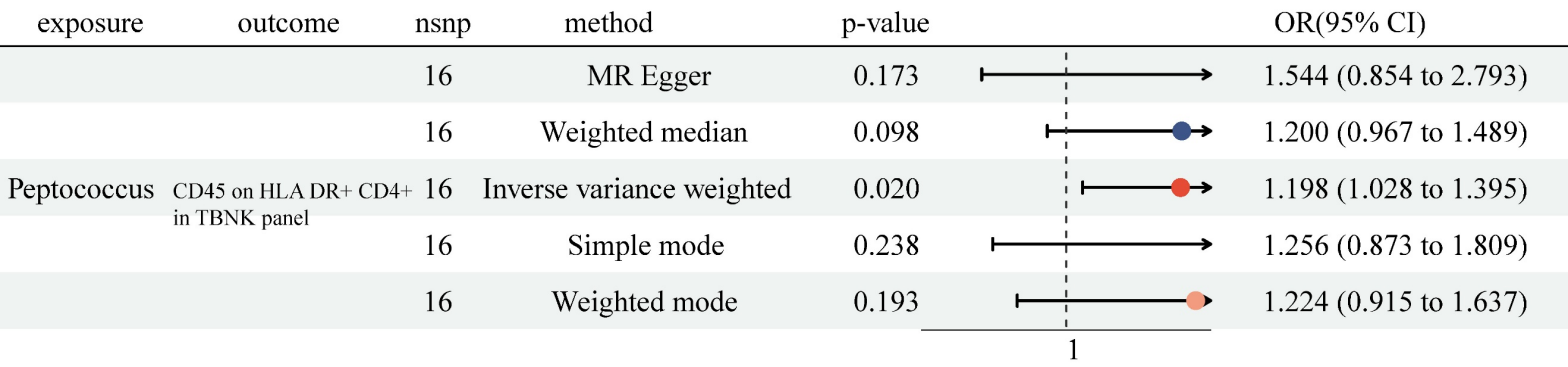
The MR analysis showed Genus-Peptococcus was highly associated with CD45 on HLA DR^+^ CD4^+^ in TBNK panel. CI: Confidence Interval; GM: Gut microbiota; MR: Mendelian randomization; OR: odds ratio; SNP: Single nucleotide polymorphism.

Previously, we identified 6 genera and 38 immune cell traits vital to NSCLC. Then, we investigated the causal role of 6 genera on 38 immune cell traits. The MR analysis revealed that only Genus-Peptococcus was highly associated with CD45 on HLA DR^+^ CD4^+^ (OR = 1.198, 95% CI [1.028, 1.395], P = 0.020, Figure 4 and Table S4). At the same time, no significant causal effect was determined with other approaches (MR Egger: (OR = 1.544, 95% CI [0.854, 2.793], P = 0.173); Weighted median: (OR = 1.200, 95% CI [0.967, 1.489], P = 0.098); Simple mode: (OR = 1.256, 95% CI [0.873, 1.809], P = 0.238); Weighted mode: (OR = 1.224, 95% CI [0.915, 1.637], P = 0.193)). No heterogeneity and horizontal pleiotropy were observed, and a particular SNP did not drive causal estimates (Table S6, Table S7 and Figure S3).

### A reverse MR analysis

**Figure 5:**
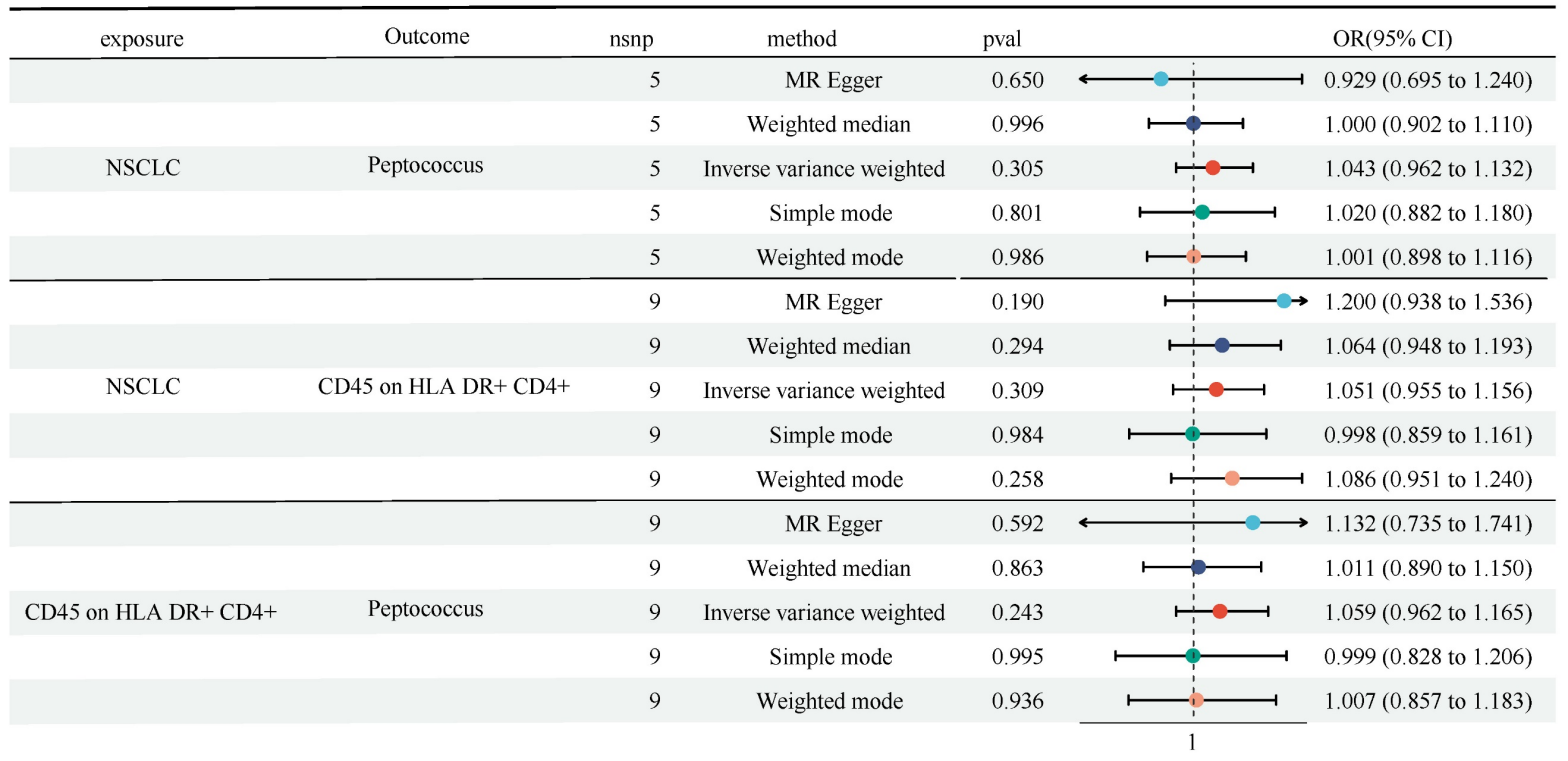
A reverse MR analysis showed no causal role of NSCLC on Genus-Peptococcus and CD45 on HLA DR^+^ CD4^+^ in TBNK panel, as well as CD45 on HLA DR^+^ CD4^+^ in TBNK panel on Genus-Peptococcus. CI: Confidence Interval; GM: Gut microbiota; MR: Mendelian randomization; OR: odds ratio; SNP: Single nucleotide polymorphism.

We found the causal role of Genus-Peptococcus and CD45 on HLA DR^+^ CD4^+^ in TBNK panel on NSCLC, and the role of Genus-Peptococcus on CD45 on HLA DR^+^ CD4^+^. Next, we performed a reverse MR analysis. Figure 5 showed that no obvious causal effect of NSCLC on Genus-Peptococcus (OR = 1.043, 95% CI [0.962, 1.132], P = 0.305) and CD45 on HLA DR^+^ CD4^+^ (OR = 1.051, 95% CI [0.955, 1.156], P = 0.309) was detected (Figure 5 and Table S5). Also, no causal role of Genus-Peptococcus on CD45 on HLA DR^+^ CD4^+^ was witnessed (OR = 1.059, 95% CI [0.962, 1.165], P = 0.243). No heterogeneity and horizontal pleiotropy were discovered (Table S6 and Table S7).

### Mediation effect of GM on NSCLC

We analyzed the causal effect of Genus-Peptococcus on NSCLC and CD45 on HLA DR^+^ CD4^+^ in the TBNK panel. We performed a mediation analysis to depict the mediation effect of CD45 on HLA DR^+^ CD4^+^ between Genus-Peptococcus on NSCLC. The mediation effect of CD45 on HLA DR^+^ CD4^+^ in the causal pathway from Genus-Peptococcus to NSCLC was -034 (95% CI [-0.070, -0.005]; P = 0.037, Table 1), accounting for 14.4% of the total effect.

## Discussion

Over the last ten years, many studies have come to understand the significance of the gut microbiota in the pathophysiological processes of various diseases, including LC 8. Previously, numerous studies have shown that compared to healthy individuals, the composition and structure of GM were significantly altered in NSCLC patients, hinting that GM may contribute to the occurrence and development of NSCLC 9. To investigate the causal effect of GM on NSCLC, we performed a two-sample MR. We found the causality of 6 genera on NSCLC was significant (Bacteroides, Roseburia, Alistipes, Methanobrevibacter, Ruminococcus gauvreauii group, and Peptococcus). Regulation of the immune system is one of the essential ways in which GM plays a biological role. To explore whether those 6 genera regulate the immune system to affect NSCLC, we conducted a two-step MR. Firstly, we detected that 27 immune cell traits had protective effects on NSCLC, and 11 immune cell traits had disadvantageous effects on NSCLC. Secondly, we analyzed the causal effects of 6 genera on 38 immune cell traits. The results indicated that among 6 genera, only Genus-Peptococcus and CD45 on HLA DR^+^ CD4^+^ had close association. According to our findings, CD45 on HLA DR+ CD4+ may be an essential moderator in the causal pathway from Genus-Peptococcus to the risk of NSCLC. Additionally, we calculated the proportion of indirect effects using mediation analyses. The data showed the mediation effect of CD45 on HLA DR^+^ CD4^+^ was -034 and took over 14.4% of the total effect (P=0.037), suggesting that CD45 on HLA DR^+^ CD4^+^ was a critical mediator in the relationship between NSCLC risk and Genus-Peptococcus. However, the MR analysis revealed no causal role of Genus-Peptococcus on lung adenocarcinoma or squamous cell lung carcinoma (Table S8).

Genus-Peptococcus is a group of gram-positive coccus. The G^+^C content of its DNA is 35.7-36.7% 27. It could utilize organic nutrients through fermentation metabolism and produce H_2_ from peptone without utilizing carbohydrates 28. Studies illustrated that the abundance of Genus-Peptococcus in several cancers was significantly decreased, like prostate cancer and gastric cancer, and was closely related to cancer cachexia 29-31. However, so far, there is no research indicating whether there are differences in the composition and distribution of Genus-Peptococcus in NSCLC.

Animal experiments have shown that regulating GM, including Genus-Peptococcus, could enhance host immune function 32,33. Herein, we demonstrated that CD45 on HLA DR^+^ CD4^+^ in the TBNK panel may be a critical moderator between Genus-Peptococcus and NSCLC. CD45 is widely present on the surface of white blood cells, and its cytoplasmic region acts as a protein tyrosine phosphatase, which can dephosphorylate and activate tyrosine on substrates P56lck and P59fyn 34. CD45 is a critical molecule in signal transduction on the cell membrane and plays a vital role in the development, maturation, functional regulation, and signal transmission of lymphocytes, which is highly related to tumor immunization 35,36.

In the study, we investigated the causal role of GM on NSCLC with an MR design and the mesomeric effect of immune cells between the association of GM and NSCLC. In an observational setting, this study could simulate randomized controlled trials with a lower cost and less risk of reverse causal effect. However, there were still some limitations that should be acknowledged. 1) The potential heterogeneity and horizontal pleiotropy cannot be fully assessed. 2) The generalizability of the conclusion was restricted as all data were gathered from the European population; it is necessary to validate the conclusions in other populations. 3) We collected NSCLC cases from public database. The sample size of NSCLC was only 1627, which may bias the results. 4) The pathway from Genus-Peptococcus to NSCLC was found to be partly mediated by CD45 on HLA DR^+^ CD4^+^ in the TBNK panel. However, the mediation effect was -034, only accounting for 14.4% of the total effect. Other mediators may also exist and require further attention.

## Conclusion

Our study illustrated the causal relationships between GM, immune cells, and NSCLC. Specifically, Genus-Peptococcus could decrease the risk of NSCLC, which was, to a large proportion, mediated by CD45 on HLA DR^+^ CD4^+^ in the TBNK panel.

## Supplementary Material

Supplementary figures.

## Figures and Tables

**Table 1:** Mediation effect of Genus-Peptococcus on NSCLC via CD45 on HLA DR^+^ CD4^+^ in TBNK panel.

Total effect	Direct effect A	Direct effect B	Mediation effect	P	Mediated Proportion (%)
β (95% CI)	β (95% CI)	β (95% CI)	β (95% CI)		
-0.236 (-0.330 to -0.142)	0.180 (0.103 to 0.257)	-0.189 (-0.229 to -0.149)	-0.034 (-0.070 to -0.005)	0.037	14.44

Total effect: The causal role of GM on NSCLC. Direct effect A: The causal role of GM on immune cell traits. Direct effect B: The causal role of immune cell traits on NSCLC. β(indirect effect) = β(Direct effect A) * β(Direct effect B). The mediated proportion = β(indirect effect) / β(total effect).

## Abbreviations

AC: Absolute cell
CI: Confidence Interval
GM: Gut microbiota
GWAS: genome-wide association studies
LC: Lung cancer
NSCLC: Non-small cell lung cancer
MFI: median fluorescence intensities
MP: morphological parameters
MR: Mendelian randomization
MR-PRESSO: MR pleiotropy residual sum and outlier method
OR: odds ratio
RC: relative cell
SNP: Single nucleotide polymorphism
IVs: Instrumental variables
IVW: Inverse variance weighted
TBNK: B cells, natural killer cells, T cells
