# Supplementary material for: Causal relationships between gut microbiota, immune cell, and Non-small cell lung cancer: a two-step, two-sample Mendelian randomization study
Source: J Cancer. 2024 Feb 4;15(7):1890–7. doi: 10.7150/jca.92699 (PMC10905411; doi:10.7150/jca.92699)
Supplement: Supplementary file 1 — Supplementary figures. [file jcav15p1890s1.pdf]

Figure S1. The leave-one-out sensitivity analysis of the MR analysis of 6 genera on NSCLC.

Figure S2. The leave-one-out sensitivity analysis of the MR analysis of immune cells on NSCLC.

Figure S3. The leave-one-out sensitivity analysis of the MR analysis of Genus-Peptococcus on CD45 on HLA DR^+ $CD4^+$ and the reverse MR analysis.

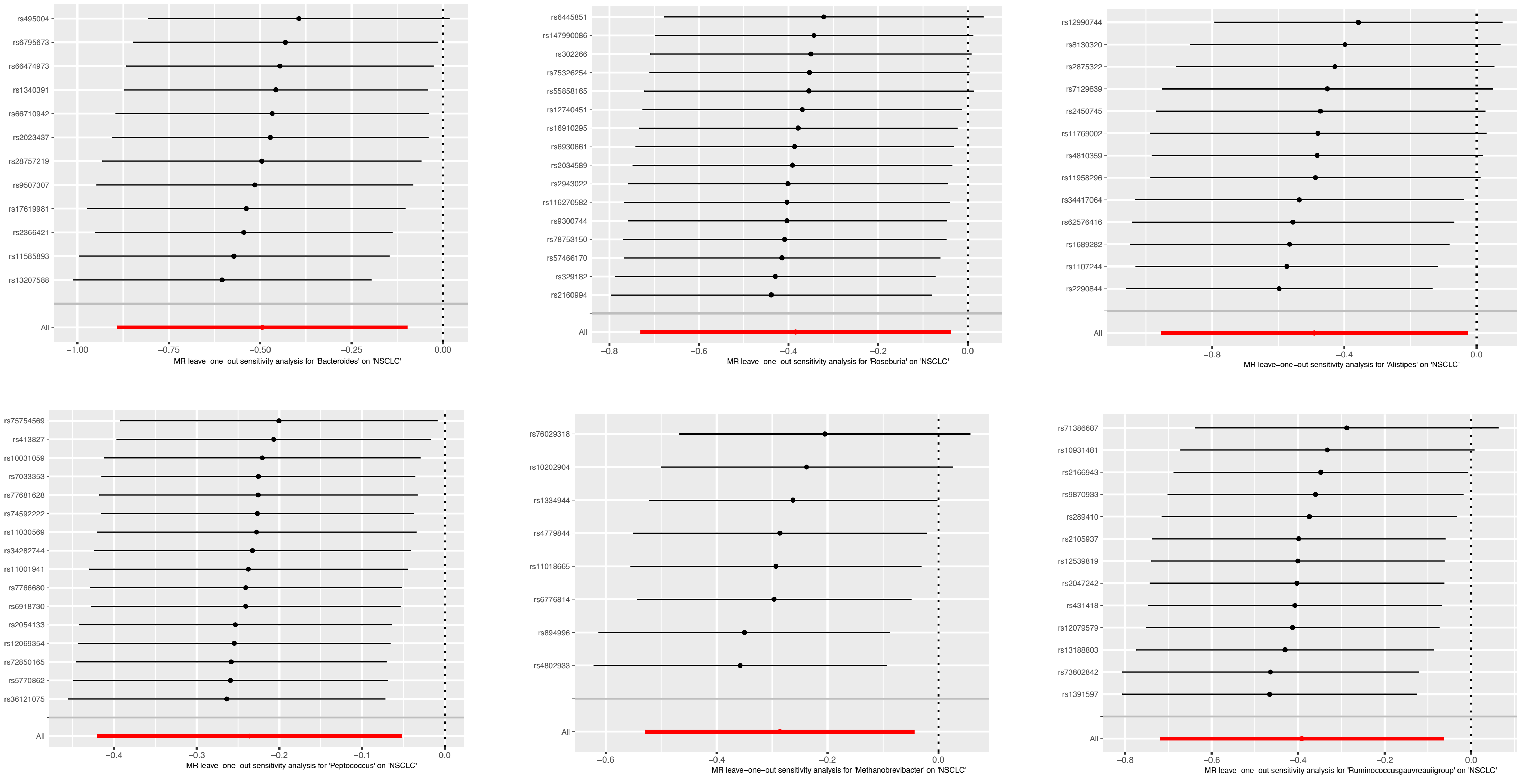
rs495004
rs6795673
rs66474973
rs1340391
rs66710942
rs2023437
rs28757219
rs9507307
rs17619981
rs2366421
rs11585893
rs13207588
All
−1.00
−0.75
−0.50
−0.25
0.00
MR leave−one−out sensitivity analysis for 'Bacteroides' on 'NSCLC'
rs6445851
rs147990086
rs302266
rs75326254
rs55858165
rs12740451
rs16910295
rs6930661
rs2034589
rs2943022
rs116270582
rs9300744
rs78753150
rs57466170
rs329182
rs2160994
All
−0.8
−0.6
−0.4
−0.2
0.0
MR leave−one−out sensitivity analysis for 'Roseburia' on 'NSCLC'
rs12990744
rs8130320
rs2875322
rs7129639
rs2450745
rs11769002
rs4810359
rs11958296
rs34417064
rs62576416
rs1689282
rs1107244
rs2290844
All
−0.8
−0.4
0.0
MR leave−one−out sensitivity analysis for 'Alistipes' on 'NSCLC'
rs75754569
rs413827
rs10031059
rs7033353
rs77681628
rs74592222
rs11030569
rs34282744
rs11001941
rs7766680
rs6918730
rs2054133
rs12069354
rs72850165
rs5770862
rs36121075
All
−0.4
−0.3
−0.2
−0.1
0.0
MR leave−one−out sensitivity analysis for 'Peptococcus' on 'NSCLC'
rs76029318
rs10202904
rs1334944
rs4779844
rs11018665
rs6776814
rs894996
rs4802933
All
−0.6
−0.4
−0.2
0.0
MR leave−one−out sensitivity analysis for 'Methanobrevibacter' on 'NSCLC'
rs71386687
rs10931481
rs2166943
rs9870933
rs289410
rs2105937
rs12539819
rs2047242
rs431418
rs12079579
rs13188803
rs73802842
rs1391597
All
−0.8
−0.6
−0.4
−0.2
0.0
MR leave−one−out sensitivity analysis for 'Ruminococcusgauvreauiigroup' on 'NSCLC'

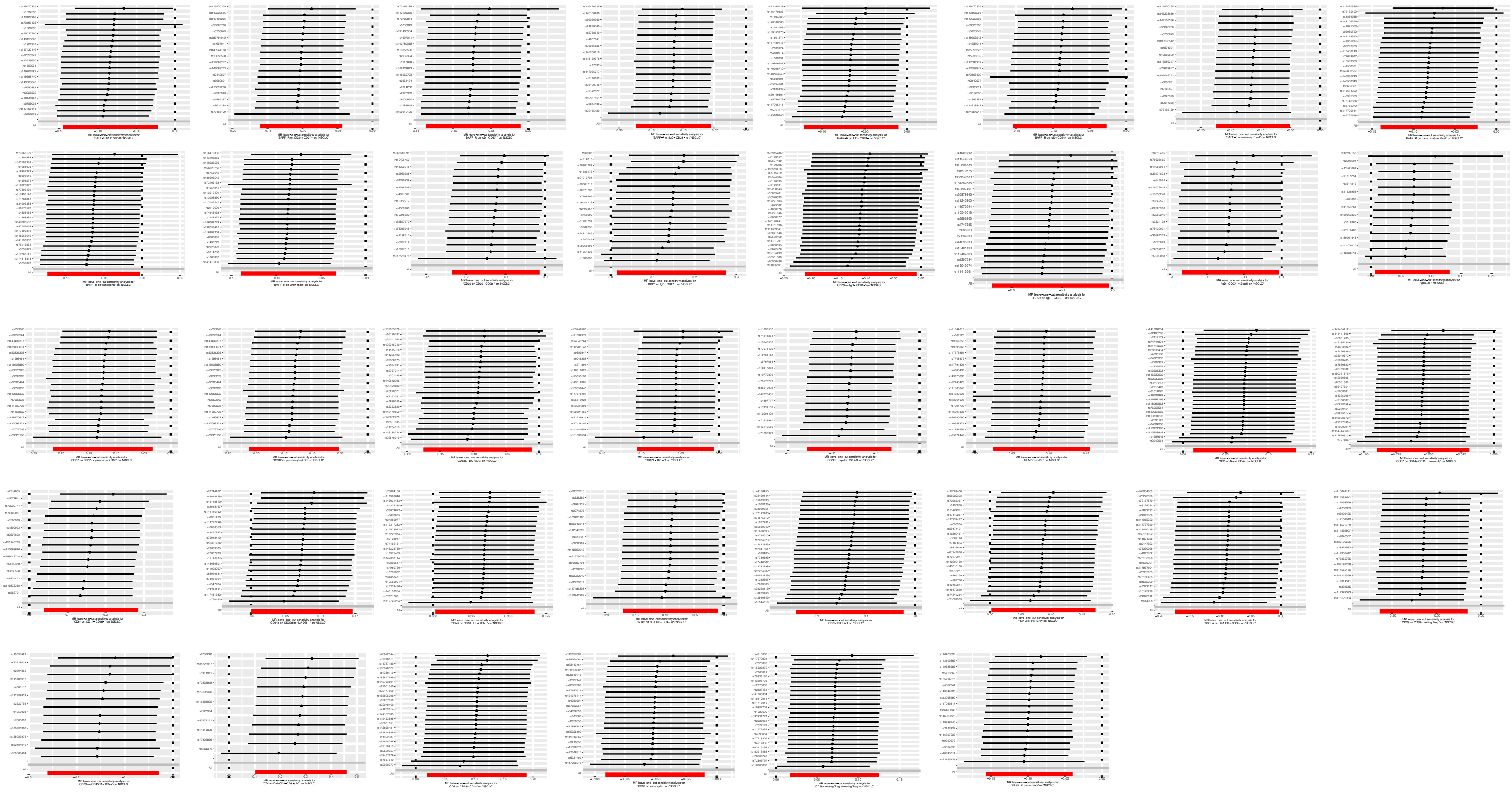

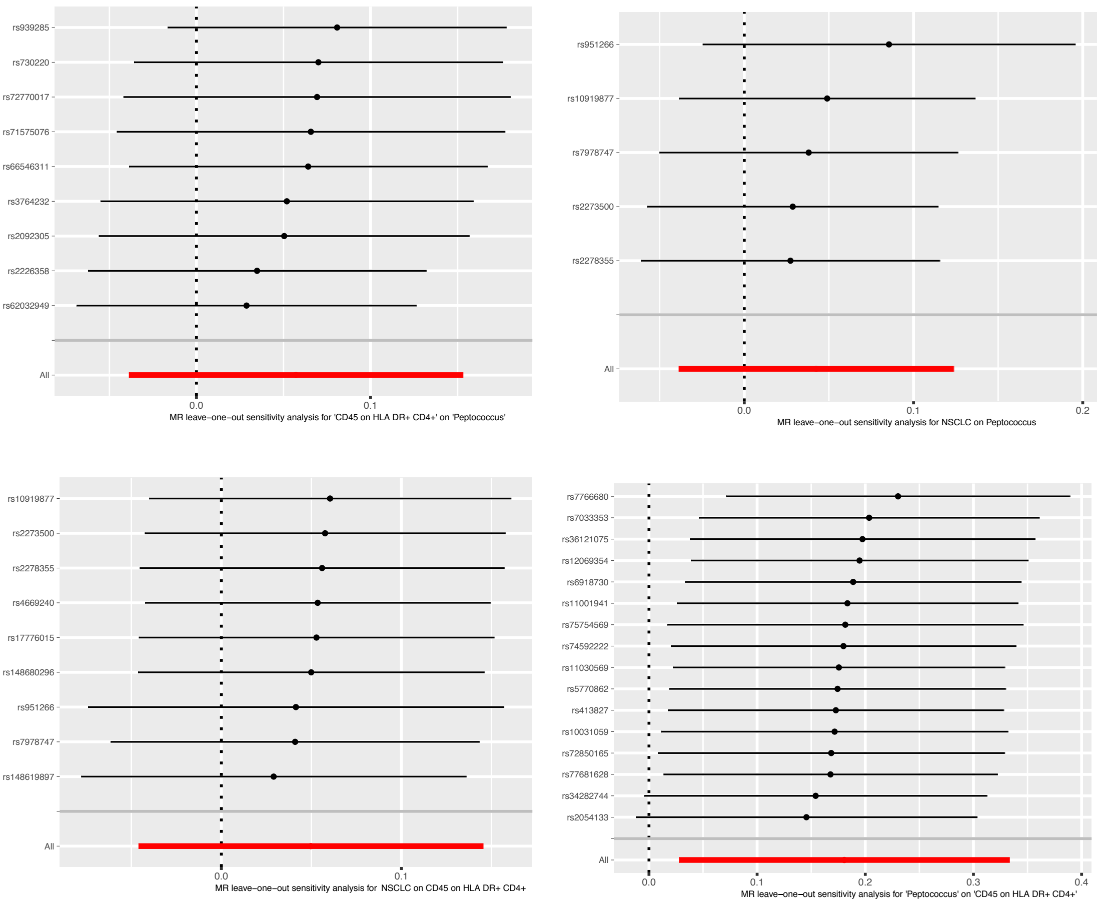
rs939285
rs730220
rs72770017
rs71575076
rs66546311
rs3764232
rs2092305
rs2226358
rs62032949
All
0.0
0.1
MR leave−one−out sensitivity analysis for 'CD45 on HLA DR+ CD4+' on 'Peptococcus'
rs951266
rs10919877
rs7978747
rs2273500
rs2278355
All
0.0
0.1
0.2
MR leave−one−out sensitivity analysis for NSCLC on Peptococcus
rs10919877
rs2273500
rs2278355
rs4669240
rs17776015
rs148680296
rs951266
rs7978747
rs148619897
All
0.0
0.1
MR leave−one−out sensitivity analysis for NSCLC on CD45 on HLA DR+ CD4+
rs7766680
rs7033353
rs36121075
rs12069354
rs6918730
rs11001941
rs75754569
rs74592222
rs11030569
rs5770862
rs413827
rs10031059
rs72850165
rs77681628
rs34282744
rs2054133
All
0.0
0.1
0.2
0.3
0.4
MR leave−one−out sensitivity analysis for 'Peptococcus' on 'CD45 on HLA DR+ CD4+'
